# Implicating bites from a leishmaniasis sand fly vector in the loss of tolerance in pemphigus

**DOI:** 10.1172/jci.insight.123861

**Published:** 2020-12-03

**Authors:** Soumaya Marzouki, Ines Zaraa, Maha Abdeladhim, Chaouki Benabdesselem, Fabiano Oliveira, Shaden Kamhawi, Mourad Mokni, Hechmi Louzir, Jesus G. Valenzuela, Melika Ben Ahmed

**Affiliations:** 1 Laboratory of Transmission, Control and Immunobiology of Infections, LR11IPT02, Pasteur Institut de Tunis, Tunis, Tunisia.; 2 Department of Dermatology, La Rabta Hospital Tunis, Tunis, Tunisia.; 3 Faculty of Medicine de Tunis, University of Tunis El Manar, Tunis, Tunisia.; 4 Vector Molecular Biology Section, Laboratory of Malaria and Vector Research, National Institute of Allergy and Infectious Diseases (NIAID), NIH, Rockville, Maryland, USA.

**Keywords:** Autoimmunity, Immunology, Autoimmune diseases, Tolerance

## Abstract

A possible etiological link between the onset of endemic pemphigus in Tunisia and bites of *Phlebotomus papatasi*, the vector of zoonotic cutaneous leishmaniasis, has been previously suggested. We hypothesized that the immunodominant *P*. *papatasi* salivary protein PpSP32 binds to desmogleins 1 and 3 (Dsg1 and Dsg3), triggering loss of tolerance to these pemphigus target autoantigens. Here, we show using far-Western blot that the recombinant PpSP32 protein (rPpSP32) binds to epidermal proteins with a MW of approximately 170 kDa. Coimmunoprecipitation revealed the interaction of rPpSP32 with either Dsg1 or Dsg3. A specific interaction between PpSP32 and Dsg1 and Dsg3 was further demonstrated by ELISA assays. Finally, mice immunized with rPpSP32 twice per week exhibited significantly increased levels of anti-Dsg1 and -Dsg3 antibodies from day 75 to 120. Such antibodies were specific for Dsg1 and Dsg3 and were not the result of cross-reactivity to PpSP32. In this study, we demonstrated for the first time to our knowledge a specific binding between PpSP32 and Dsg1 and Dsg3, which might underlie the triggering of anti-Dsg antibodies in patients exposed to sand fly bites. We also confirmed the development of specific anti-Dsg1 and -Dsg3 antibodies in vivo after PpSP32 immunization in mice. Collectively, our results provide evidence that environmental factors, such as the exposure to *P*. *papatasi* bites, can trigger the development of autoimmune antibodies.

## Abstract 2

A specific binding between *Phlebotomus papatasi* salivary protein PpSP32 and desmogleins may trigger anti-Dsg antibodies in patients exposed to sand fly bites.

## Introduction

Pemphigus is an organ-specific autoimmune bullous disease characterized by keratinocyte detachment, resulting from the presence of autoantibodies targeting 2 major desmoglein (Dsg) proteins, Dsg1 and Dsg3, approximately 160 and 130 kDa, respectively (1). Similar to other autoimmune diseases, pemphigus is the consequence of the interaction between predisposing genetic factors and inducing environmental triggers. However, events triggering the development of anti-Dsg antibodies are still largely unknown.

Tunisian pemphigus shares clinical and epidemiological features with Fogo Selvagem, an endemic form of pemphigus foliaceous in Brazil. Several findings point to the involvement of vector-borne parasite infections, particularly leishmaniasis, in the pathogenesis of endemic pemphigus (2, 3). In Tunisia, zoonotic cutaneous leishmaniasis (ZCL) attributed to *Leishmania major* (*L*. *major*) is more prevalent in rural areas where a higher incidence of Tunisian endemic pemphigus has been reported (4). Such areas are also characterized by high densities of *Phlebotomus papatasi* (*P*. *papatasi*), the vector of ZCL. Such findings may suggest the involvement of sand fly bites — specifically the injected insect salivary proteins — in triggering the disease. Our recent results support such a hypothesis as an increase in the frequency and level of antibodies directed against salivary proteins of *P*. *papatasi* was demonstrated in patients with pemphigus compared with healthy subjects (5). Such antibodies are essentially composed of the IgG4 isotype and directed against the commonly recognized salivary proteins of *P*. *papatasi*, more particularly against the salivary protein, PpSP32, the immunodominant protein recognized by all subjects naturally exposed to the bites of this vector (5–7). A persistent exposure to *P*. *papatasi* bites may, therefore, be a possible etiologic link to pemphigus in Tunisia.

Here, we demonstrate that during the bite of *P*. *papatasi*, a direct interaction between the immunogenic salivary protein PpSP32 and desmogleins 1 and 3 (Dsg1 and Dsg3) triggers the production of anti-Dsg antibodies, possibly leading to the development of this autoimmune disease.

## Results

### Far-Western blot shows interaction of the recombinant PpSP32 protein with epidermal proteins of about 170 kDa.

**Figure 1:**
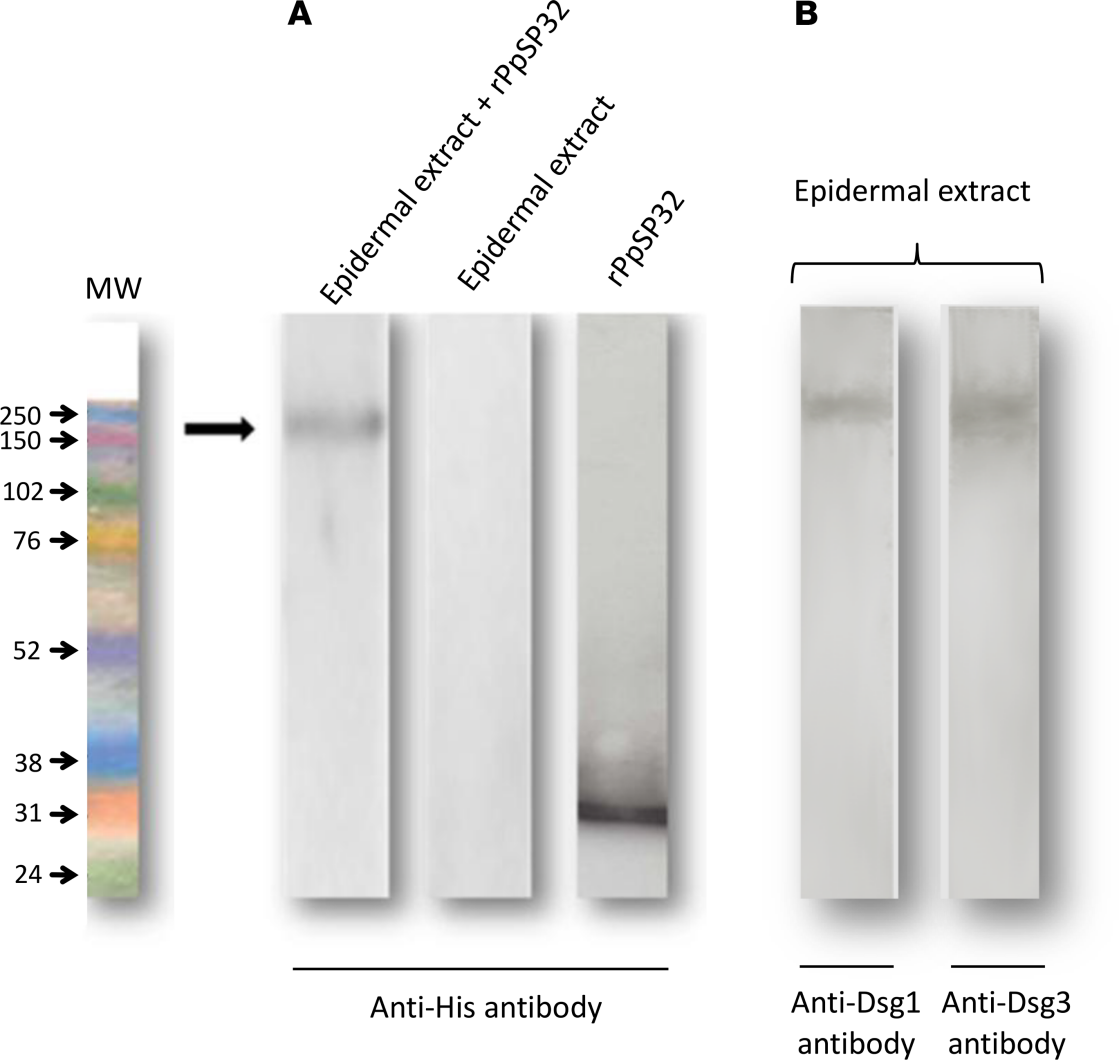
Binding of rPpSP32 to an epidermal protein of approximately 170 kDa demonstrated by a far-Western blot. The proteins of an epidermal extract were separated by PAGE. After being denatured and renatured, the proteins were either not incubated or incubated with the recombinant protein rPpSP32. (**A**) The putative interaction of rPpSP32 with 1 or more epidermal proteins was revealed using a peroxidase-labeled anti-His antibody. Controls correspond to the epidermal extract or the rPpSP32 His-tagged protein alone. The figure is representative of 3 independent experiments. (**B**) Dsg1 and Dsg3 were visualized in the epidermal extract using monoclonal anti-Dsg1 and -Dsg3 antibodies. MW marker, RPN 800E (Amersham), was used in this experiment.

A far-Western blot technique was used to test a possible interaction between the sand fly rPpSP32 protein and human epidermal proteins. Epidermal proteins were extracted from skin samples and separated on a polyacrylamide gel. After transfer to a nitrocellulose membrane, the proteins were denatured and then renatured using decreasing doses of guanidine-HCl and then incubated in the presence of the his-tagged recombinant PpSP32 protein (rPpSP32). An anti-polyhistidine (anti-His) antibody revealed a band of approximately 170 kDa indicative of a binding of the rPpSP32 to human epidermal proteins (Figure 1A). There was no direct binding between the anti-His antibody and the epidermal extract, and as expected, the rPpSp32 alone was visualized at the 32 kDa MW range (Figure 1A). Dsg1 and Dsg3 proteins were visualized in the epidermal extract using mAbs re vealing bands at the 170 kDa MW range (Figure 1B).

### Coimmunoprecipitation revealed that rPpSP32 interacts with Dsg1 and Dsg3.

**Figure 2:**
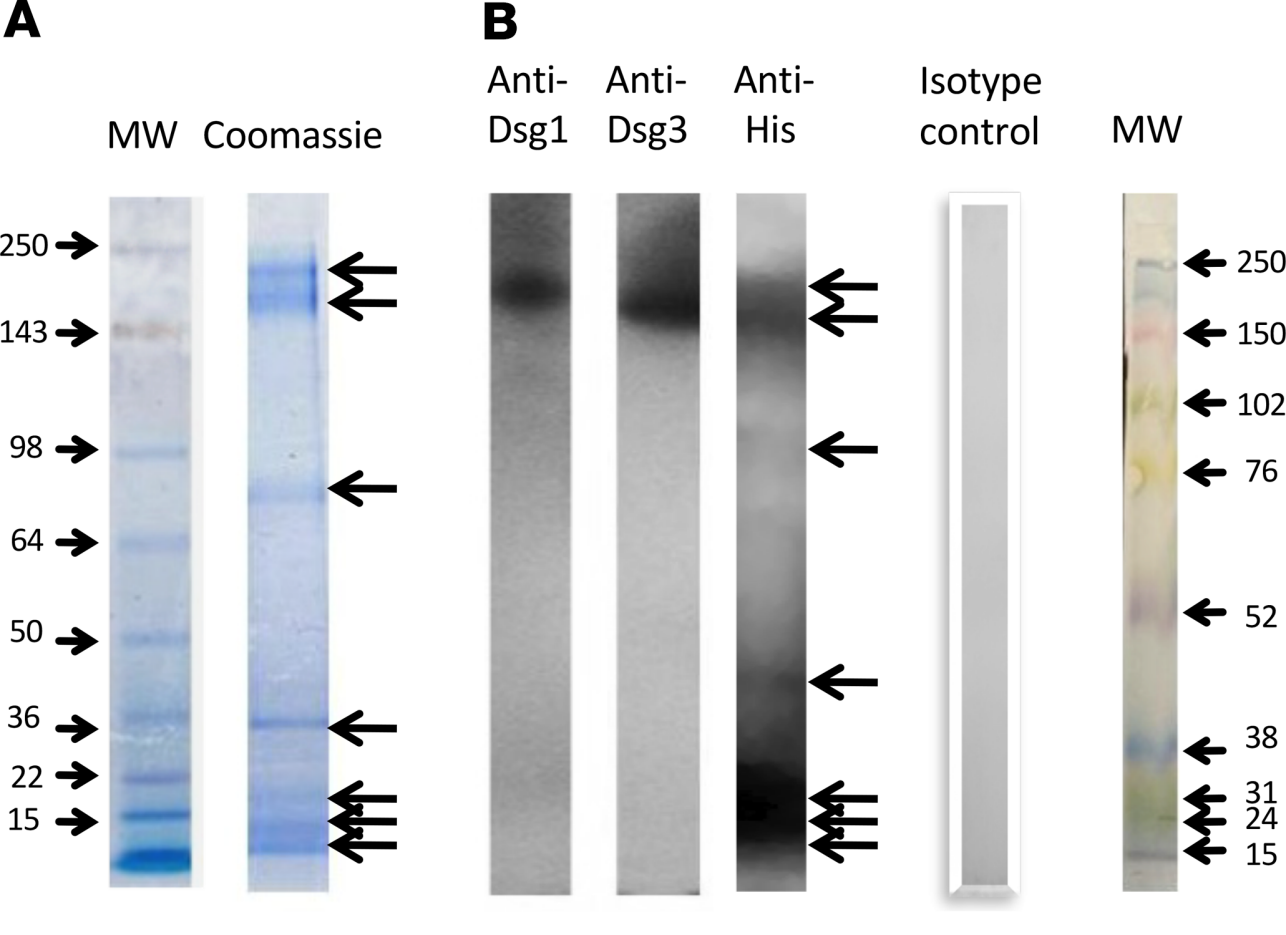
Identification of binding partners of PpSP32 by coimmunoprecipitation. The mixture of the epidermal extract and rPpSP32 was coimmunoprecipitated in the presence of magnetic beads adsorbed with anti-polyhistidine (anti-His) capture antibody. The coimmunoprecipitation (Co-IP) eluate was separated by SDS-PAGE and then analyzed by Coomassie blue staining (**A**) and Western blotting using monoclonal anti-human Dsg1, anti-human Dsg3, anti-His antibodies, or isotype control (**B**). The figure is representative of 3 independent experiments.

To confirm the interaction of PpSP32 with human epidermal proteins, specifically Dsg1 and/or Dsg3 proteins, we performed a coimmunoprecipitation from a mixture of rPpSP32 and the epidermal extract in the presence of magnetic beads sensitized with anti-His capture antibody. As shown in Figure 2A, proteins that bound to the anti-His antibodies were run on an SDS-PAGE and stained by Coomassie blue. We observed that 2 proteins with a MW of approximately 150 and 170 kDa were precipitated, in addition to the rPpSP32. Notably, PpSP32 frequently forms a pattern of several secondary bands as previously demonstrated (6). We confirmed the identity of the high-MW-precipitated bands as Dsg1 and Dsg3 by its specific reactivity on an immunoblot against anti-human Dsg1 and anti-Dsg3 mAbs (Figure 2B). Moreover, we also tested the precipitate against anti-His antibody, observing that the binding of PpSP32 with both Dsg1 and Dsg3 (high-MW bands) was maintained during the SDS-PAGE (Figure 2B).

### rPpSP32 interacts specifically with Dsg1 and Dsg3 in a dose-dependent manner.

**Figure 3:**
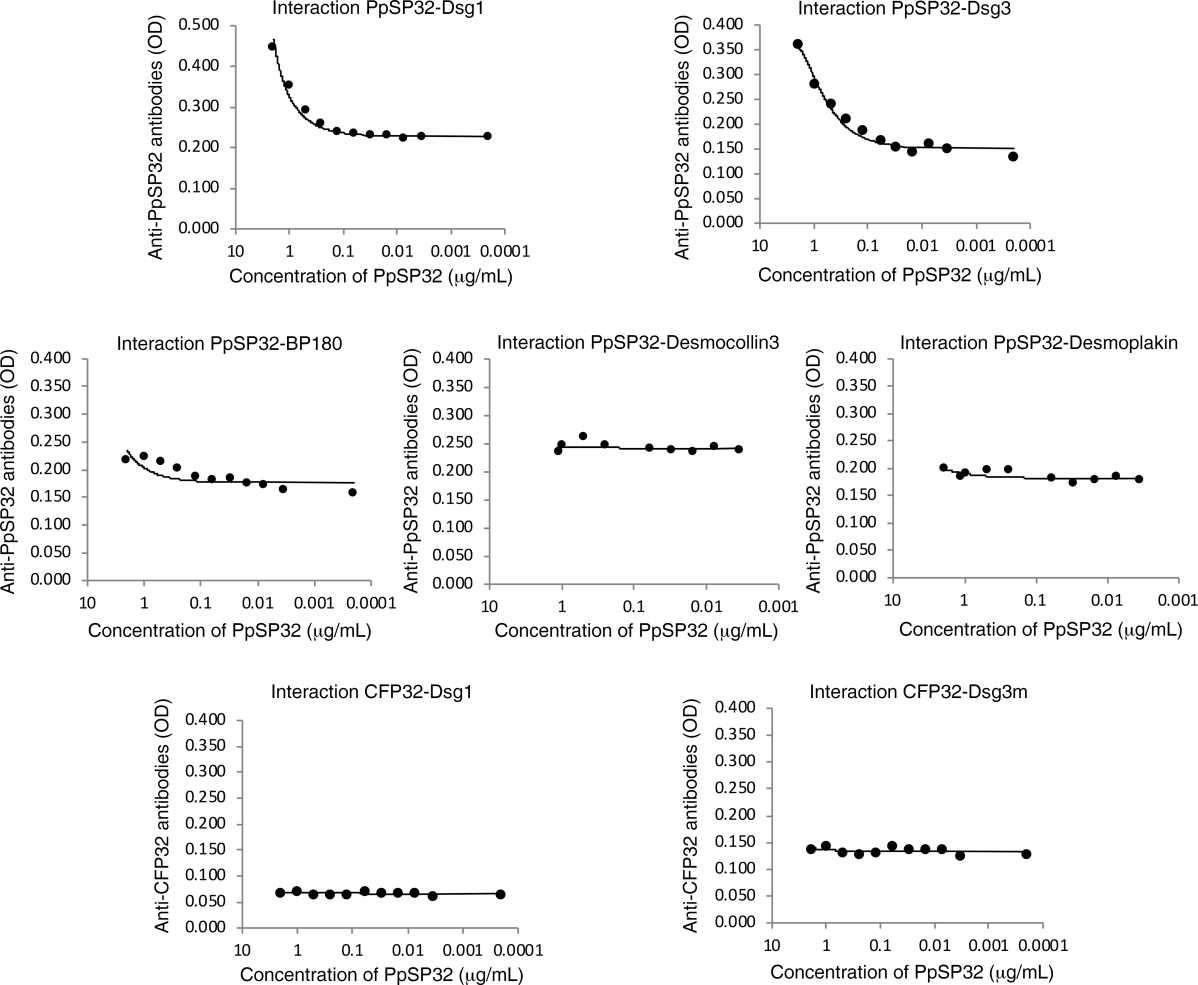
ELISA showing a specific and dose-dependent interaction between PpSP32 and the proteins Dsg1 and Dsg3. The ELISA plates were coated with the recombinant proteins Dsg1 and Dsg3 or other epidermal proteins; BP180, desmocollin 3 and desmoplakin and then incubated with a decreasing concentration of rPpSP32, the protein of interest or CFP32, a control protein. The plates were then incubated in the presence of human sera with anti-PpSP32 or -CFP32 antibodies. The results are representative of 3 independent experiments. Significant differences were found between the different PpSP32 concentrations for Dsg1 and Dsg3 (*P* = 0.006 and *P* = 0.008 between the condition 2 μg/mL and 0.0002 μg/mL for Dsg1 and Dsg3, respectively). For the control proteins, no significant difference was obtained while decreasing the concentration of PpSP32 (*P* > 0.05).

To further confirm the specificity of the interaction between rPpSP32 protein and Dsg1 and Dsg3, ELISA plates were coated with the recombinant human proteins Dsg1 and Dsg3. The plates were then incubated with a decreasing concentration of rPpSP32, then the binding of PpSP32 was tested after several washes using previously tested sera containing anti-PpSP32 antibodies. In additional experiments, 4 control proteins were used, including BP180, the immunodominant target in bullous pemphigoid (BP), desmocollin 3 and desmoplakin, 2 proteins of the desmosomes (as control proteins for Dsg1 and Dsg3), and an irrelevant control protein of similar MW as PpSP32 (recombinant form of the CFP32 protein of *Mycobacterium tuberculosis* [8]) as a protein control for PpSP32). A specific and dose-dependent interaction between rPpSP32 and Dsg1 and Dsg3 was observed (Figure 3). No interaction was observed between rPpSP32 and BP180, desmocollin 3, or desmoplakin or between CFP32 and the Dsgs (Figure 3).

### Mice immunized with PpSP32 develop antibodies specific for Dsg1 and Dsg3.

**Figure 4:**
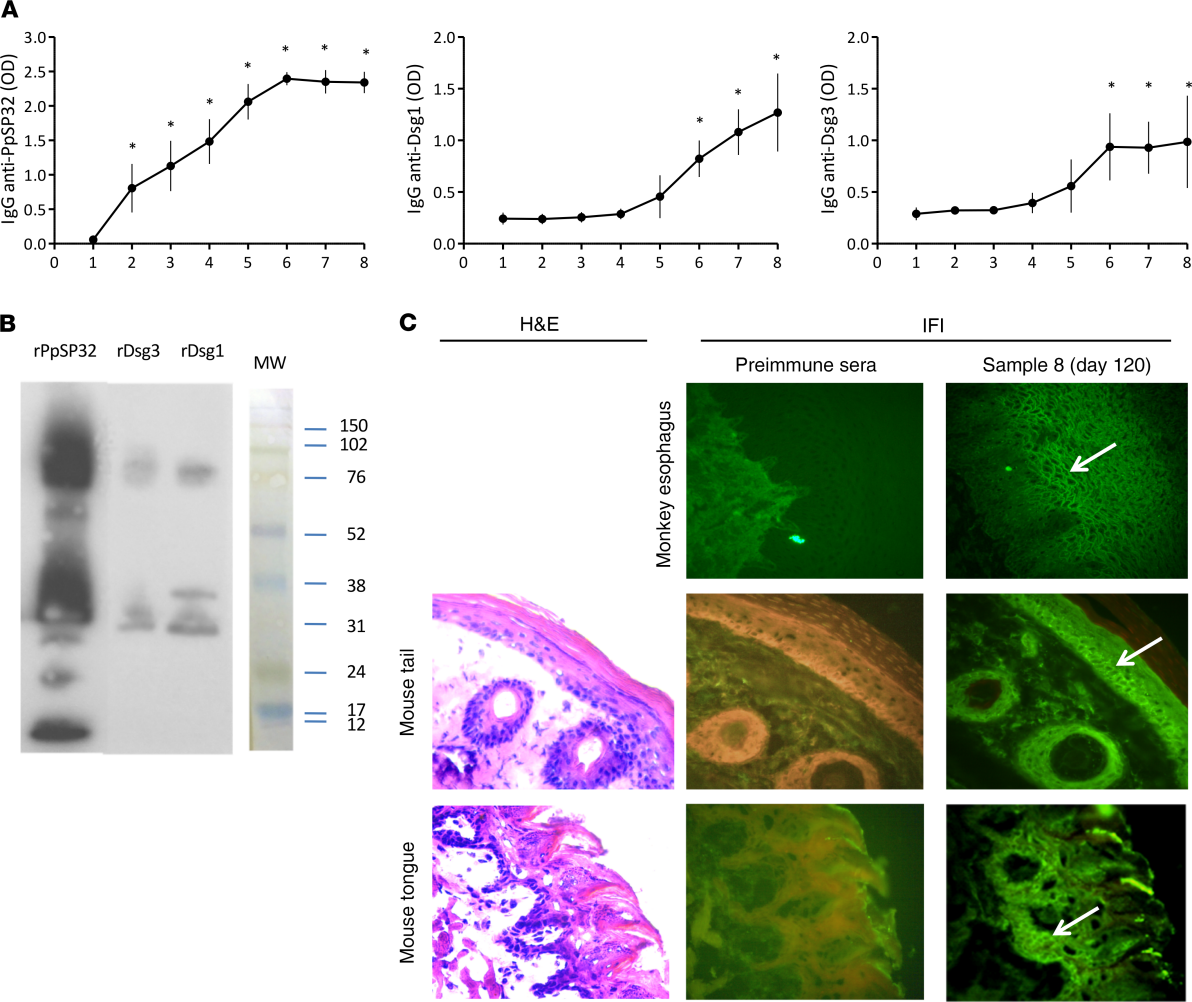
Development of anti-Dsg1 and -Dsg3 antibodies in mice immunized with PpSP32. Mice were immunized intradermally with 2 μg rPpSP32 twice weekly over 30 days and then once per week for 90 days. (**A**) The sera, recovered every 15 days (samples 1–8) were tested by ELISA to detect total IgG antibodies anti-PpSP32, -Dsg1, and -Dsg3. *P < 0.05. Student’s *t* test when compared with sample 1. For anti-Dsg1 antibodies, *P* = 0.009, *P* = 0.01, and *P* = 0.01 at the sixth, seventh, and eighth sample, respectively. For anti-Dsg3 antibodies, *P* = 0.008, *P* = 0.025, and *P* = 0.0016 at the sixth, seventh, and eighth sample, respectively. (**B**) Sera recovered at day 120 (sample 8) were tested by Western blot against rPpSP32, partial Dsg1 (rDsg1, MW 77 KDa), or partial Dsg3 (rDsg3, MW 79 KDa). The result is representative of 3 independent experiments. (**C**) Preimmune sera and sera recovered at day 120 (sample 8) were tested by immunofluorescence on monkey esophagus substrate. A representative photo is shown. Original magnification, ×40. (**D**) Preimmune sera and sera recovered at day 120 (sample 8) were tested by immunofluorescence on monkey esophagus substrate and mouse tail and tongue biopsies. Representative photos for H&E staining and indirect immunofluorescence (IFI) are shown. Original magnification, ×40.

To confirm in vivo the implication of PpSP32 in the development of antibodies linked to pemphigus, Swiss Webster mice were immunized intradermally with 2 μg rPpSP32 over 4 months. The sera were recovered every 15 days and tested for the presence of both anti-PpSP32 antibodies and anti-mouse Dsg1 and Dsg3 antibodies. As shown in Figure 4A, mice immunized with rPpSP32 developed, as expected, anti-PpSP32 antibodies at high titers. Interestingly, such mice also developed anti-Dsg1 and -Dsg3 antibodies at high levels that were significant (*P* < 0.05) from the sixth sampling (at day 90) to the eighth sampling (at day 120) when compared with those at the first sampling after immunization. Western blot analyses confirmed the development of such specific antibodies (Figure 4B). When tested by immunofluorescence on monkey esophagus and mouse tail and tongue, such antibodies gave the characteristic pattern of intercellular substance staining, further confirming recognition of the Dsgs compared with tissues tested with preimmune sera (Figure 4C).

**Figure 5:**
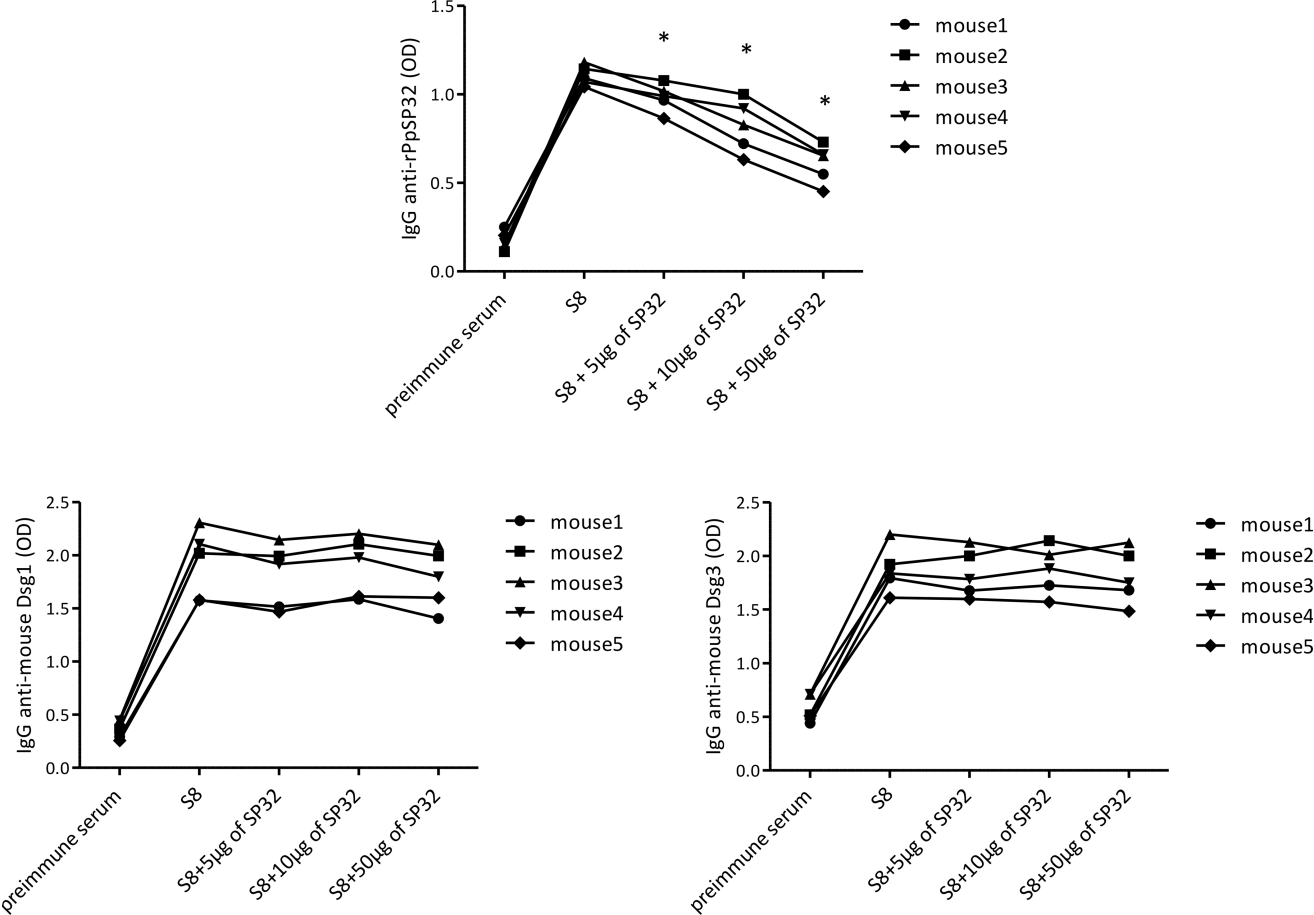
Anti-Dsg1 and -Dsg3 antibodies did not result from a cross-reactivity with PpSP32. The preimmune sera as well as sample 8 (recovered at day 120) of mice immunized with rPpSP32 were either not preincubated or preincubated with increasing concentrations of rPpSP32 and then tested by ELISA to detect anti-PpSP32, -Dsg1, or -Dsg3 antibodies. *P < 0.05. Student’s *t* test used when compared with S8 (for IgG anti-PpSP32, *P* = 0.0052, *P* = 0.0072, and *P* = 0.0002 for the conditions with PpSP32 at 5 μg/mL, 10 μg/mL, and 50 μg/mL, respectively).

Finally, to test whether the developed anti-mouse Dsg1 and anti-Dsg3 antibodies cross-react with PpSP32, a competition assay was performed in which the mouse sera were first preincubated with rPpSP32 at increasing concentrations and then tested against Dsg1 or Dsg3. As shown in Figure 5, although preincubating sera with rPpSP32 impacted the recognition of PpSP32, this had no effect on the recognition of Dsg1 and Dsg3.

### People exposed to P. papatasi bites develop specific antibodies for Dsg1 and Dsg3 the titers of which are correlated with those of anti-PpSP32 antibodies.

**Figure 6:**
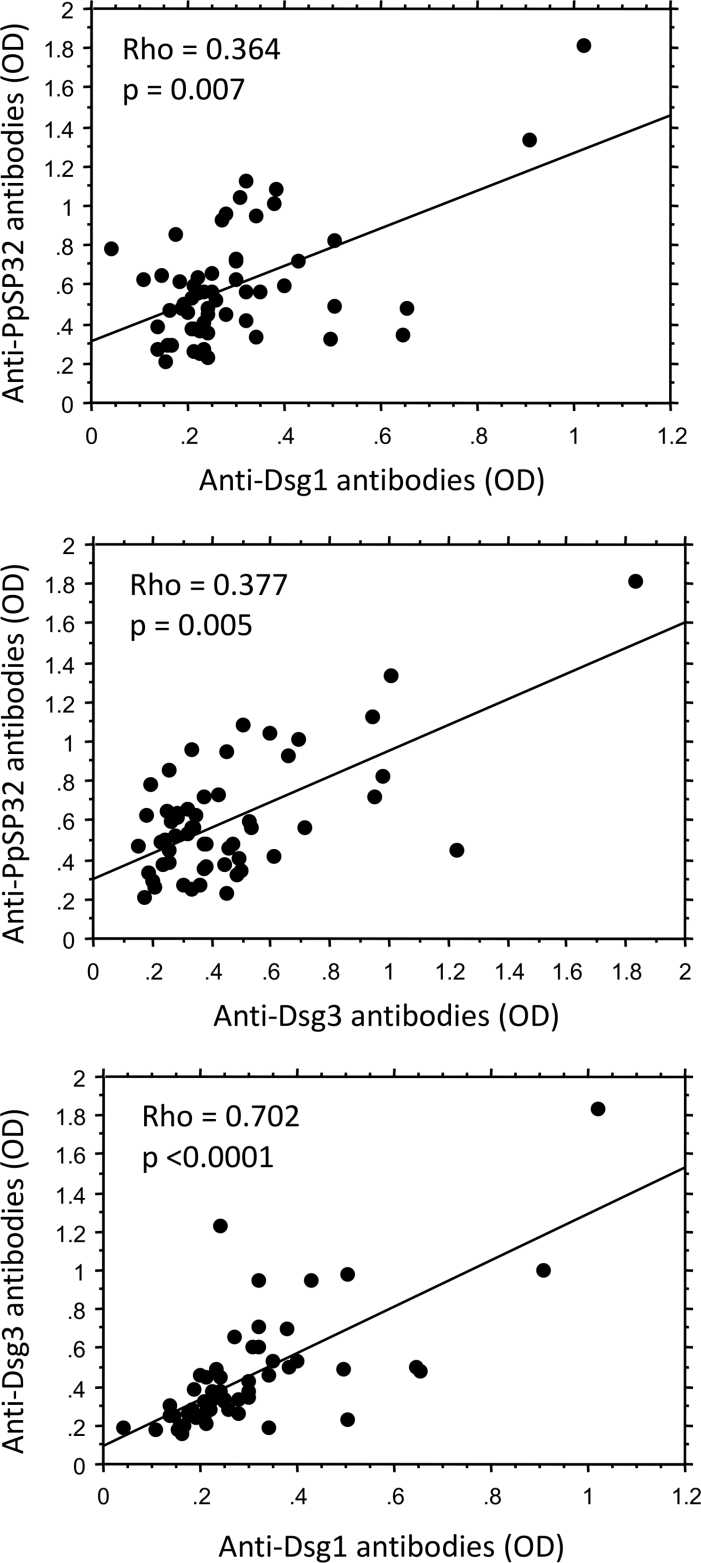
Correlations between the levels of anti-PpSP32, -Dsg1, and -Dsg3 antibodies in donors naturally exposed to *P. papatasi* bites. Specific IgG antibodies against rPpSP32, human Dsg1, and Dsg3 were quantified by ELISA in sera of 56 donors naturally immunized against the saliva of *P*. *papatasi* and exhibiting anti-Pp32 antibodies with levels covering an OD range of positivity from 0.28 to 1.82. Correlation analyses were performed using Spearman’s rank test.

We previously demonstrated that the prevalence of anti-Dsg1 antibodies was significantly higher in ZCL patients living in endemic areas compared with healthy controls from the same regions (13.3% vs. 1.6%) (5).Here, we quantified anti-PpSP32, anti-human Dsg1, and anti-human Dsg3 antibodies in 72 representative donors selected from a previous population survey consisting of 522 donors living in endemic areas for ZCL caused by *L*. *major* and characterized by the presence of *P*. *papatasi* at high frequencies (9). Notably, none of these subjects were diagnosed with endemic pemphigus at the time of the sera collection. Our data showed a relatively high prevalence of anti-Dsg1 and -Dsg3 antibodies in the donors exhibiting anti-PpSP32 antibodies. Accordingly, among the 56 donors with positive anti-PpSP32 antibodies, 14 were positive for anti-Dsg1 and 17 were positive for anti-Dsg3 antibodies. Interestingly, the titers of anti-Dsg1 and -Dsg3 antibodies significantly correlated with those of anti-PpSP32 antibodies (*P* = 0.007 and *P* = 0.005, respectively) (Figure 6). Again, preincubating sera with rPpSP32 had no effect on Dsg1 and Dsg3 recognition (data not shown).

## Discussion

Pemphigus is the most common autoimmune bullous dermatosis in Tunisia (10). In southern and central areas, pemphigus foliaceus occurs in its endemic form (6.7 cases/million/year) mainly in young women and is more prevalent in rural areas with a high contact with ruminants (11). It also shares several features with Fogo Selvagem, the endemic form of the Brazilian pemphigus. In Brazil, case-control studies suggest that certain living conditions, including exposure to blood-feeding insect bites, may constitute risk factors for endemic pemphigus. Accordingly, epidemiological data indicated a significant association between pemphigus and exposure to black flies (12–14). Similarities between Dgs1 and salivary proteins of black fly *Simulium nigrimanum* has even been suggested (15). Moreover, patients with leishmaniasis, Chagas disease, and onchocerciasis frequently have anti-Dsg1 antibodies (2, 16–18).

Comparable data have been accumulated in Tunisia where pemphigus is endemic. In an epidemiological case-control study, several environmental factors such as wasp, bee, and spider stings have been shown to be significantly associated with pemphigus (11). Further, it has been demonstrated that anti-Dsg1 and -Dsg3 antibodies were more prevalent in subjects with visceral leishmaniasis (3). Moreover, in our previous case-control study, patients with pemphigus had significantly higher titers of antibodies against salivary gland extracts of *P*. *papatasi*, the vector of zoonotic cutaneous leishmaniasis (5). Such antibodies were directed particularly against the immunodominant target of PpSP32 (5, 6). This suggests that persistent exposure to *P*. *papatasi* bites and an intense antibody immune response to PpSP32 could be a trigger to the autoimmune response in pemphigus.

To test this hypothesis, we first used a far-Western technique and demonstrated that rPpSP32 binds to 1 or more epidermal proteins with a MW of approximately 170 kDa, which may correspond to Dsg1 and/or Dsg3. To better identify such proteins, we performed a coimmunoprecipitation of the mixture between an epidermal extract and rPpSP32 using an anti-His capture antibody. Therefore, we showed that rPpSP32 coimmunoprecipitated with 2 MW proteins of about 150 and 170 kDa. Subsequent use of the anti-Dsg1 and -Dsg3 mAbs confirmed that the bound proteins were actually Dsg1 and Dsg3. To better define the specificity of the interaction between Dsgs and the immunodominant salivary protein rPpSP32, we subsequently performed an ELISA test in which the microtiter plates were coated with the recombinant proteins Dsg1, Dsg3, BP180, desmocollin 3, or desmoplakin, other epidermal proteins, and then incubated with rPpSP32 or a control protein (CFP32). Such experiments demonstrated that rPpSP32 interacts specifically and in a dose-dependent manner with Dsg1 and Dsg3, whereas no interaction was demonstrated between rPpSP32 and BP180, desmocollin 3, or desmoplakin or between Dsg proteins and CFP32. We also investigated the interaction of PpSP32 with Dsgs by using surface Plasmon resonance analysis (data not shown). Results showed a weak signal and provided data could not be fitted, precluding determination of the kinetic parameters. Using the whole molecule of Dsg induced a molecular crowding that prevented the molecules from moving freely because of the sterically hindered structure of Dsg, which lead to a blockage of the protein absorbance.

Finally, to further support the implication of PpSP32 in the development of specific pemphigus antibodies in vivo, Swiss Webster mice were immunized intradermally with rPpSP32. Sera collected periodically for 3 months revealed the expected development of anti-PpSP32 antibodies at high titers. Interestingly, such mice developed from the sixth sample (day 90) either had anti-Dsg1 and/or -Dsg3 antibodies with significant high titers. Yet, anti-Dsg1 antibodies seem to be more prominently developed than anti-Dsg3 antibodies as shown by the Western blot analysis. The staining pattern obtained by immunofluorescence on monkey esophagus and mouse tail and tongue biopsies further confirms the specificity of recognition of the intercellular substance. Slight differences in the pattern of immunofluorescence obtained in the different tissues may be owing to the variable ratio of Dsg1/Dsg3. Collectively, the results obtained in mice are close to those obtained by the Brazilian team of Diaz et al., which suggest the involvement of another salivary protein LJM11 of *Lutzomyia longipalpis*, a New World vector of leishmaniasis, in the loss of tolerance in Brazilian pemphigus (19, 20). The authors showed that, in an endemic area of leishmaniasis and pemphigus in Brazil, IgG4 antibodies directed against Dsg1 of patients with pemphigus foliaceous recognize LJM11. In addition, mice immunized with this salivary protein produce anti-Dsg 1 antibodies (19, 20). Such results strongly support that LJM11 induces antibodies that cross-react with Dsg1. Importantly, a cross-reactivity between antibody responses against Dsg1 and LJM11 was recently demonstrated (21). The presence of cross-reactive antibodies in pemphigus indicates the presence of at least 1 immunogenic conformational epitope, which is overlapping between the environmental antigen and the autoantigen (21). Remarkably, Diaz and colleagues showed more recently that a high proportion of mice immunized with the *L*. *longipalpis* LJM17 salivary protein developed cross-reactive antibodies against human Dsg1 (22).

The hypothesis of a cross-reaction between anti-PpSP32 and -Dsg1 or -Dsg3 antibodies has been tested using a competition assay. Our results showed that preincubating sera of immunized mice with rPpSP32 did not abolish the recognition of Dsg1 or Dsg3, supporting the specificity of the developed antibodies towards Dsg1. Therefore, our data are in favor of protein-protein interaction between PpSP32 and Dsg1 and Dsg3, a hypothesis further supported by data obtained in silico. In fact, by using I-TASSER/Zhang and PyMOL V0.9/CAUCH software and servers, we showed that PpSP32 was able to bind to Dsgs by involving the cadherin domains EC1 and EC2 (Supplemental Figure 1; supplemental material available online with this article; https://doi.org/10.1172/jci.insight.123861DS1).

**Figure 7:**
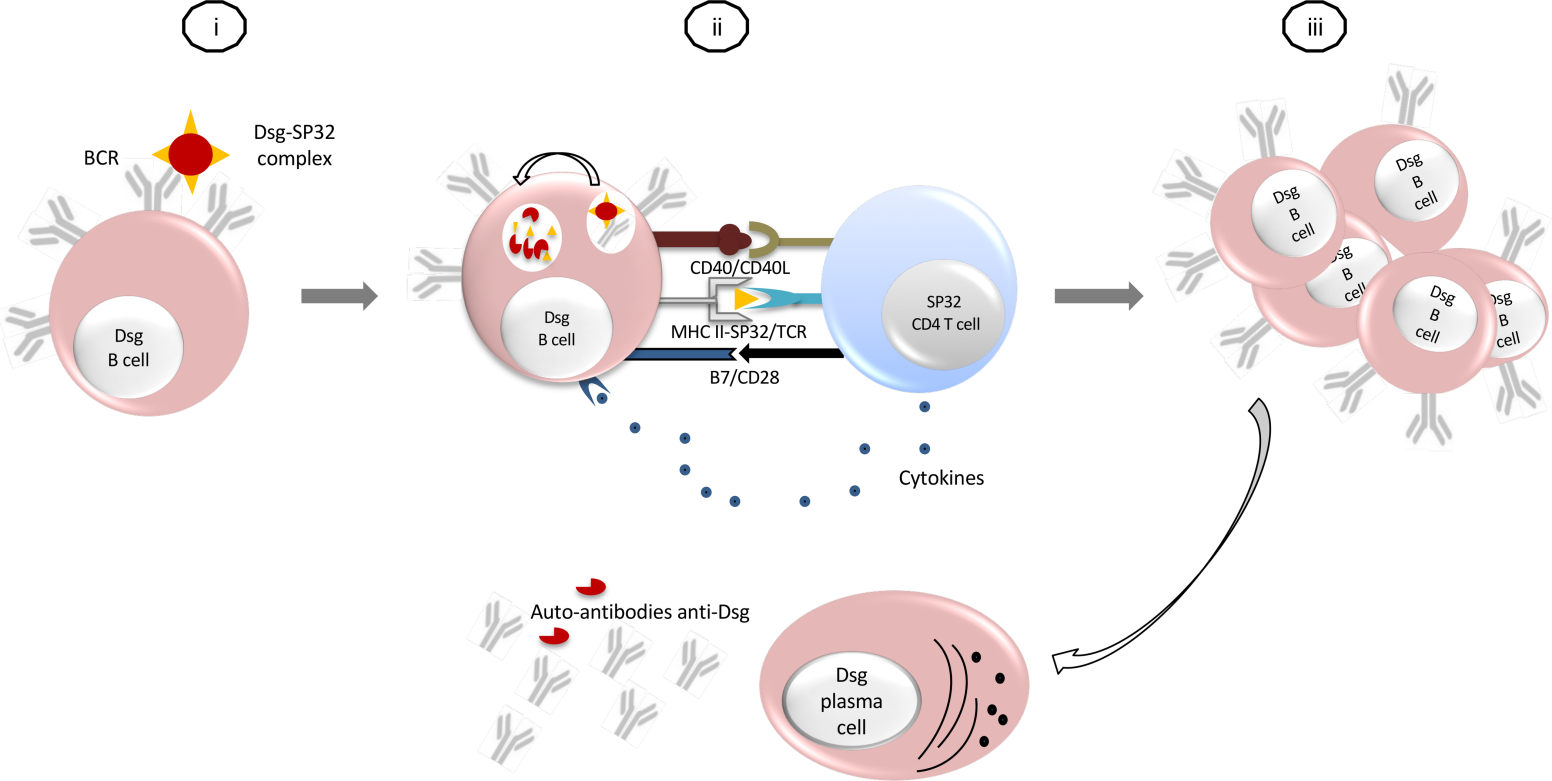
Hypothetic model explaining how anti-Dsg antibodies are induced after exposure to SP32 sand fly salivary protein. (i) The complex PpSP32-Dsgs are recognized by Dsg-reactive B cells. (ii) Dsg-reactive B cells endocytose and process the Dsg-PpSP32 complex. They present the PpSP32 peptides to the specific SP32-reactive T cells. Stimulated T cells give the necessary help (cytokines secretion and CD40 ligation) to specific Dsg B lymphocytes. (iii) Dsg B cells proliferate and differentiate into plasma cells producing anti-Dsg autoantibodies.

Our results demonstrate for the first time to our knowledge the interaction of the salivary protein of *P*. *papatasi*, PpSP32, with the epidermal proteins Dsg1 and Dsg3. PpSP32 is a puzzling and intriguing protein whose function is unknown to date. It harbors structural homologies with other proteins such as a flagelliform silk protein of *Nephila clavipes* (23). Interestingly, it also has homologies with collagen adhesion proteins and is predicted to be a mucin based on the pattern of its O- and N-glycosylation (24). Thus, its interaction with epidermal proteins, namely Dsgs, is very likely and explains how immunization against PpSP32 could trigger the production of anti-Dsg antibodies leading to the development of pemphigus. Such a mechanism has been already involved in other autoimmune pathologies, particularly in celiac disease in which the interaction between gluten, the alimentary protein, and tissue transglutaminase, the autoantigen, leads to the loss of tolerance against the latter in genetically predisposed subjects (25). Therefore, we suggest that following the exposure to *P*. *papatasi* bite and the binding of PpSP32 to Dsgs, specific Dsg B cells would endocytose and process the Dsg-PpSP32 complex then present the PpSP32 peptides to the PpSP32-reactive T cells. The latter, thus activated, will give the necessary help to the specific Dsg-B lymphocytes in order to activate and induce the production of anti-Dsg antibodies (Figure 7).

Our results support that an environmental component may induce or at least initiate the autoimmune response in pemphigus. Notably, the anti-Dsg1 and -Dsg3 antibodies induced in our immunized mice do not seem to be pathogenic as suggested by the absence of bullous lesions. Additional genetic factors are probably essential for the induction of pathogenic antibodies. Thus, exposure to the salivary antigens of *P*. *papatasi* could only play an initiating role in the activation of an autoimmune antibody response. Accordingly, several studies indicate that HLA and non-HLA susceptibility genes are involved in the triggering of pemphigus foliaceous in Tunisia, and that healthy subjects expressing nonpathogenic anti-Dsg1 antibodies are protected by the presence of specific alleles (26, 27).

Other data obtained in humans also support the involvement of salivary antigens of *P*. *papatasi* in triggering anti-Dsg antibody development. Accordingly, we and others previously demonstrated that the prevalence of anti-Dsg antibodies was significantly higher in either visceral or cutaneous leishmaniasis compared with healthy controls from the same regions (3, 5). Interestingly, individuals with high titers anti-PpSP32 antibodies are more at risk of becoming ZCL probably due to a higher exposure to *P*. *papatasi* bites (6). Here, we showed a high prevalence of anti-Dsg1 and -Dsg3 antibodies in people naturally immunized against PpSP32. Remarkably, the titers of such antibodies were correlated with those of anti-SP32 antibodies, further suggesting the involvement of PpSP32 exposure in triggering anti-Dsg antibody development.

Collectively, our results provide evidence that environmental factors, such the exposure to *P*. *papatasi* bites, could trigger the development of pathogenic autoantibodies and the development of pemphigus in genetically predisposed subjects. We thus establish a clear relationship between a noninfectious antigen of the environment and the development of potentially pathogenic autoantibodies in an autoimmune disease. Using several techniques, we demonstrated a specific binding between PpSP32 and Dsg1 and Dsg3 and confirmed in vivo the development of specific anti-Dsg antibodies after PpSP32 immunization. In perspective, we are seeking to solve the structure of PpSP32 after crystallization to gain further insight into the molecular interactions involved in its binding to Dsg proteins.

## Methods

### Human epidermal protein extract.

Protein extraction from the human epidermis was carried out according to the Stanley technique (27). Skin samples of mammectomy were defatted and cut into shreds of approximately 1 mm × 1 cm. The epidermis was separated from the dermis using forceps after incubation of the flaps in a 1 M NaCl solution overnight with stirring at room temperature. The epidermal sheets were weighed and then transferred to a lysis buffer supplemented with 3 mL protease inhibitors (Roche Diagnostics GmbH) per 1 g tissue. After 1-hour incubation at room temperature, the epidermis was ground with an Ultra-Turrax T8 high-pressure homogenizer (IKA) for 30 seconds then placed on ice and sonicated using a Vibra Cell ultrasound apparatus (Fisher Bioblock Scientific) for 15 cycles of 9 seconds of ultrasound followed by 9 seconds of shutdown. The preparations obtained were then heated at 100°C for 5 minutes and centrifuged at 13,000*g* for 30 minutes. The supernatant was aliquoted and stored at –80°C. The protein extract was assayed using Bradford reagent (MilliporeSigma). The absorbance was measured at a wavelength equal to 595 nm. After each preparation, the extract was tested by analytical electrophoresis on a polyacrylamide gel.

### Expression and purification of rPpSP32.

Total RNAs were extracted from 1- to 2-day-old female sand fly salivary glands using the RNeasyMini Kit (Qiagen). The extracted RNA was then reverse transcribed using the Moloney murine leukemia virus reverse transcriptase (Invitrogen) and random hexamers (Promega) according to a standard procedure. The PpSP32-specific cDNA was amplified using a forward primer deduced from the amino-terminus sequence (starting after the signal peptide) and a reverse primer encoding a hexahistidine motif. The PCR product was inserted into an expression vector, plasmid VR-TOPO2010, according to the procedure described previously (28) and sent to the Frederick National Laboratory for Cancer Research (Frederick, Maryland, USA) for expression in HEK-293F cells. The supernatant of the transfected cells was collected after 72 hours and then purified by HPLC (7).

### Human samples.

A total of 72 sera were from representative donors selected within a previous population survey consisting of 522 donors living in 5 localities in central regions of Tunisia, which are endemic for ZCL caused by *L*. *major* and characterized by the presence of *P*. *papatasi* at high frequencies (9). These donors had no bullous skin disease. Among them, 16 tested negative for anti-PpSP32 antibodies and 56 tested positive, covering the different range of positivity.

### Far-Western blot.

The epidermal extract (~60 μg) was separated in an 8% polyacrylamide gel using a loading buffer containing 1 M Tris-HCl (pH 6.8), 10% SDS, 20% glycerol, 1 mM DTT, and 0.3% bromophenol blue. The separated proteins were then transferred to a nitrocellulose membrane and denatured and renatured with a buffer containing 5 M NaCl, 1 M Tris (pH 7.5), 0.5 M EDTA, 10% Tween 20, 2% skim milk powder, 10% glycerol, and 1 M DTT by gradually reducing the guanidine-HCL concentration from 6 to 3, 1, 0.1, and 0 M. The membrane was then saturated for 1 hour at room temperature using a PBS buffer containing 4 mM KH_2_PO_4_, 16 mM Na_2_HPO_4_, 115 mM NaCl (pH 7.4), 0.05% Tween 20, and 5% skim milk powder. Then, the membrane was either not incubated or incubated overnight at 4°C with rPpSP32 at 1 μg/mL in buffer containing 20 mM Tris (pH 7.6), 100 mM NaCl, 0.5 mM EDTA, 10% glycerol, 0.1% Tween 20, 2% skim milk powder, and 1 mM DTT. After 3 washes with PBS-Tween (PBS-T) buffer, the membranes were incubated with 1:2000 peroxidase-labeled anti-His antibody (Invitrogen) for 1 hour at room temperature. After a final wash with the PBS-T buffer, the His-tagged protein was revealed by chemiluminescence using the Prime ECL kit (Amersham). In a parallel experiment, the membranes were incubated for 2 hours at room temperature with monoclonal mouse anti-human Dsg1 or anti-human Dsg3 antibodies or control isotype at 2 μg/mL (Invitrogen). Incubation with anti-mouse IgG (1:1000, Santa Cruz Biotechnology) conjugated to peroxidase antibodies was then performed. Proteins were revealed by chemiluminescence using the Prime ECL kit (Amersham).

### Coimmunoprecipitation and Western blotting.

Coimmunoprecipitation was performed using the Dynabeads Coimmunoprecipitation kit (Thermo Fisher). Briefly, the beads (Dynabeads M-270 Epoxy) were washed with the C1 buffer provided by the kit using the DynaMag magnetic device and then adsorbed with 6 μg unlabeled anti-His antibody (MilliporeSigma) at 4°C for 1 hour. The beads (1 mg) were incubated in the presence of a mixture of skin extract (100 μg) and rPpSP32 (25 μg) at 4°C for 1 hour. After several washes, the eluate was subjected to electrophoresis on 3 different 8% polyacrylamide gels. One gel was stained with Coomassie blue for 1 hour at room temperature and then quenched in a discoloration buffer (50% methanol, 40% deionized water, and 10% acetic acid) until visualization of proteins. The other gels were transferred to nitrocellulose membranes. After saturation with a PBS-Tween-5% skim milk buffer, membranes were incubated overnight at 4°C with monoclonal mouse anti-human Dsg1 or anti-human Dsg3 antibodies or control isotype at 2 μg/mL (Invitrogen). An incubation with anti-mouse IgG (1:1000; Santa Cruz Biotechnology) conjugated to peroxidase antibodies was then performed. In another experiment, the membrane was incubated with an anti-His antibody labeled with peroxidase (1:2000; Invitrogen). Proteins were revealed by chemiluminescence using the Prime ECL kit (Amersham).

### Protein-protein interaction assays.

The 96-well microtiter plates (Nunc, Maxisorp) were coated after optimization with 0.1 μg recombinant human proteins, Dsg1 (MyBioSource MBS2010418), Dsg3 (MyBioSource MBS2010322), BP180 (MyBioSource MBS2012222), desmocollin 3 (MyBioSource MBS637447), desmoplakin (MyBioSource MBS2031176), rPpSP32, or a control protein (recombinant form of the CFP32 protein of *Mycobacterium tuberculosis*) (8) in a carbonate-bicarbonate buffer (0.1 M, pH 9.6) at 4°C overnight. The plates were then washed 3 times with PBS-T and saturated with PBS-T containing 4% BSA for 1 hour. After several washes, the plates were incubated with decreasing concentrations of rPpSP32 (from 2 to 0.0002 μg) for 1 hour at 37°C. After several washes, the plates were incubated in the presence of human sera with anti-PpSP32 (donors living in endemic areas of ZCL and naturally exposed to *P*. *papatasi* bites) or anti-CFP32 antibodies (patients with confirmed tuberculosis based on clinical symptoms, chest radiography, and sputum smear microscopic examination) diluted at 1:100 for 2 hours at 37°C. The specific binding of the conjugate to the solid phase was revealed by the addition of the chromogenic substrate, tetramethylbenzidine (TMB) (BD Biosciences). After stopping the reaction by the addition of H_2_SO_4_, the reading was carried out using a spectrophotometer (Awareness Technology, Inc.) at 450 nm wavelength.

### Mice immunization by rPpSP32 and monitoring of antibodies.

Swiss Webster mice were immunized intradermally in the ear lobe for 4 months with 2 μg rPpSP32 diluted in PBS (5 mice) or with PBS (5 mice) twice per week for 1 month followed by once per week for an additional 3 months. Sera were recovered before immunization and then every 15 days from the first day of immunization. The monitoring of anti-PpSP32 and anti-mouse Dsg1 and Dsg3 antibodies was performed in sera by ELISA. Briefly, 96-well microtiter plates (Maxisorp NUNC, MilliporeSigma) were coated with the recombinant proteins rPpSP32, mouse Dsg1 (MyBioSource MBS1423056), or mouse Dsg3 (MyBioSource MBS9423394) diluted to 2.5 μg/mL in carbonate-bicarbonate buffer (0.1 M, pH 9.6) at 4°C overnight. The plates were then washed 3 times with PBS-T and blocked for 2 hours with 4% BSA in PBS-T. Serum samples were diluted at 1:50 in BSA/PBS-T and either not incubated or incubated with increasing concentrations of rPpSP32 (5, 10, or 50 μg/mL) for 2 hours at room temperature. Sera were then added to plates in duplicates and incubated for 2 hours at 37°C. After 6 washes with PBS-T, the plates were incubated for 1 hour at 37°C with 50 μL per well of peroxidase-labeled anti-mouse IgG (Santa Cruz) diluted at 1:2000 in 1% BSA/PBS-T. After 9 washes with PBS-T, antibody-antigen complexes were visualized by adding 100 μL/well of TMB. The reaction was then stopped with 50 μL H_2_SO4 at 2 M, and the absorbance was measured at 450 nm wavelength using an automated ELISA reader (Awareness Technology, Inc.).

A Western blot analysis was also performed to highlight anti-PpSP32, -Dsg1, and -Dsg3 antibodies. Briefly, rPpSP32 and the recombinant forms of mouse Dsg1 or mouse Dsg3 were subjected to electrophoresis on 12% polyacrylamide gels in denaturing conditions. The gels were then transferred to nitrocellulose membranes. After an overnight saturation with a PBS-Tween-5% skim milk buffer, each strip was then incubated for 2 hours with the eighth bleeding samples of immunized mice diluted at 1:50. After washing, horseradish peroxidase–linked anti-mouse IgG antibody (1:10,000; MilliporeSigma) was incubated for 1 hour. Positive bands were visualized using enhanced chemiluminescence (Amersham).

Mouse sera have been also tested by indirect immunofluorescence on monkey esophagus substrate (Biosystems) as well as on mouse tongue and tail. The slides were incubated with preimmune and immune sera diluted by one-half for 30 minutes at room temperature. After several washes, the Dsg-antibody complex was revealed after an incubation with FITC-labeled anti-mouse IgG (MilliporeSigma) for 30 minutes at room temperature and visualization by a fluorescence microscope (Leica).

### Detection of human IgG anti-PpSP32, -Dsg1, and -Dsg3 antibodies by ELISA.

Specific IgG antibodies directed against rPpSP32 and the recombinant forms of human Dsg1 and human Dsg3 were measured by ELISA in sera of donors living in the endemic area of ZCL. The wells were coated overnight at 4°C with the different proteins at 2.5 μg/mL in 0.1 M carbonate-bicarbonate buffer (pH 9.6). After washing and blocking free binding sites for 2 hours at 37°C with PBS-Tween-4% BSA, human sera diluted at 1:200 were incubated for 1 hour at 37°C. After washing, peroxidase-conjugated anti-human IgG antibody (MilliporeSigma) was incubated for 1 hour at 37°C. TMB (BD Biosciences) was then used to visualize antibody-antigen complexes. The absorbance was measured at 450 nm wavelength using an automated ELISA reader. Ten negative sera were obtained from healthy controls living in sand fly–free regions. The cut-off value for the assays was the mean OD of these negative controls plus 2 SDs.

### Statistics.

Statistical analysis was performed using StatView software (version 5.0). Two-tailed Student’s *t* test was applied for comparisons between 2 different groups. Correlations between continuous variables were assessed using Spearman’s rank correlation. A *P* value of less than 0.05 was considered significant. GraphPad Prism software (version 5.01) was used for graphical displays.

### Study approval.

All animal procedures were approved by the NIAID Office of Animal Care and Use (OACU) Committee and handled in accordance to the *Guide for the Care and Use of Laboratory Animals* (National Academies Press, 2011) and with the NIH OACU Animal Research Advisory Committee guidelines. Procedures performed on human samples were approved by the ethics committee of the Pasteur Institute of Tunis (protocol no. 07-0018). For the collection of blood samples and subsequent analyses, the participants and/or parents and guardians provided written informed consent

## Author contributions

SM, IZ, JGV, and MBA designed the research studies. SM, IZ, and MA conducted the experiments. SM, IZ, and MBA acquired and analyzed the data. CB provided the reagents. SM, FO, SK, HL, JGV, and MBA wrote the manuscript.

## Supplementary Material

Supplemental data

## Version 1 10/27/2020

In-Press Preview

## Version 2 12/03/2020

Electronic publication
