# Supplementary material for: Implicating bites from a leishmaniasis sand fly vector in the loss of tolerance in pemphigus
Source: JCI Insight. 2020 Dec 3;5(23):e123861. doi: 10.1172/jci.insight.123861 (PMC7714401; doi:10.1172/jci.insight.123861)
Supplement: Supplemental data [file jciinsight-5-123861-s008.pdf]

Supplemental Figure 1

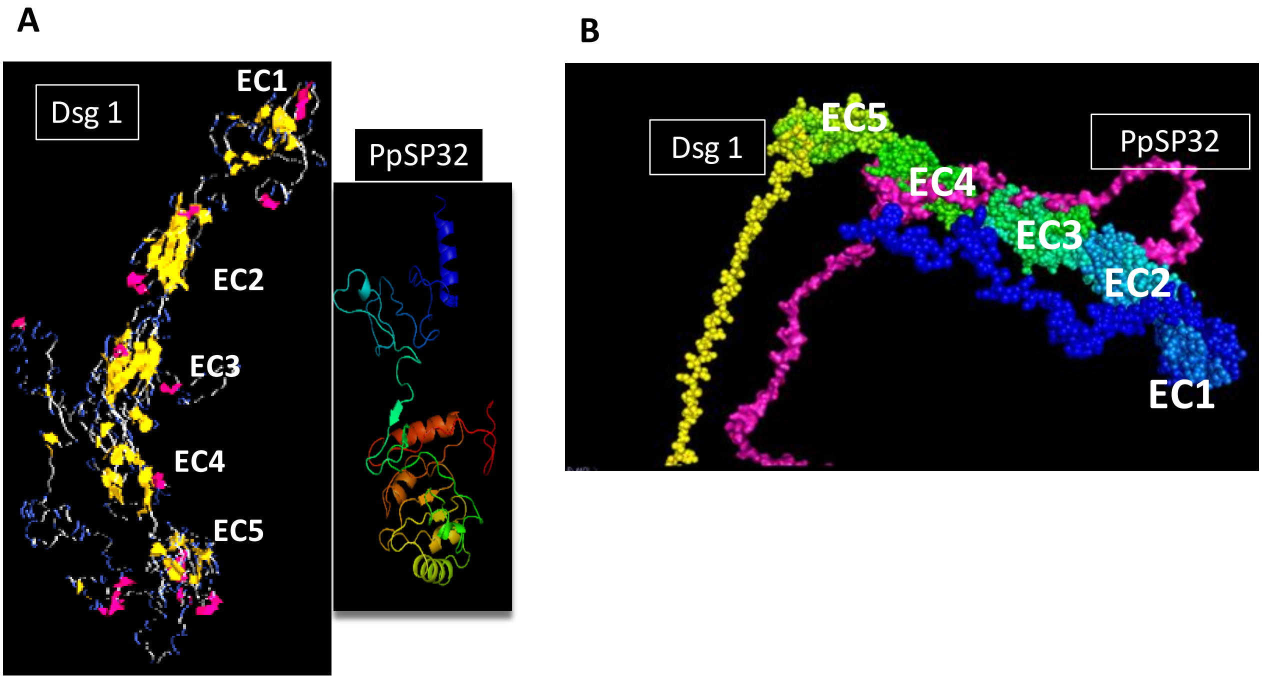

Supplemental Figure 1*: In silico* analysis of Dsg1 and PpSP32. (A) 3D structure of Dsg1 and PpSP32 predicted by I-tasser server. **(B)** *In silico* prediction of the interaction between PpSP32 (in pink and blue) and Dsg1 using PyMOL software. EC = Ectodomains.
